# The Global Prevalence of Metabolic Syndrome in Adults and Its Association With Lifestyle Factors: A Systematic Review and Meta-Analysis

**DOI:** 10.7759/cureus.105453

**Published:** 2026-03-18

**Authors:** Badrudduza Al Maimani, Faria Farhin, Shuvomoy Saha, Fahim Mahbub, Digontia Supty, Anupom Kanti Paul, Nazmin Mahezabin Tina, Fatema Binte Maksud, Nahida Sharmin, Shanta Islam

**Affiliations:** 1 Department of Internal Medicine, Mukti Hospital, Comilla, BGD; 2 Department of Nephrology, Kidney Foundation Hospital and Research Institute, Dhaka, BGD; 3 Department of Internal Medicine, Ad-din Sakina Medical College Hospital, Jashore, BGD; 4 Department of Medicine, The Barakah General Hospital, Dhaka, BGD; 5 Department of Medicine, The Barakah General Hospital Ltd., Dhaka, BGD; 6 Department of General Medicine, University Hospitals of Derby and Burton NHS Trust, Derby, GBR; 7 Department of Internal Medicine, Mymensingh Medical College Hospital, Mymensingh, BGD; 8 Department of Neonatology and Pediatric Medicine, Goyalmara Mother and Child Hospital, Cox’s Bazar, BGD; 9 Department of Internal Medicine, Popular Diagnostic Center Ltd., Dhaka, BGD; 10 Department of Medicine, Gonoshasthaya Samaj Vittik Medical College Hospital Savar, Dhaka, BGD

**Keywords:** life style, meta-analysis, metabolic syndrome, prevalence, risk factors, systematic review

## Abstract

Metabolic syndrome (MetS) is a cluster of cardiometabolic risk factors that poses a significant and growing global public health challenge. Lifestyle behaviors play a central role in its development; however, a comprehensive quantitative synthesis of global prevalence and associated lifestyle factors remains limited. This systematic review and meta-analysis aimed to estimate the pooled global prevalence of MetS in adults and examine its association with key modifiable lifestyle behaviors. Five electronic databases were systematically searched for observational studies published between 2015 and 2025 in accordance with Preferred Reporting Items for Systematic Reviews and Meta-Analyses (PRISMA) guidelines. Data were pooled using a random-effects meta-analysis. Prevalence estimates were calculated with 95% confidence intervals (CIs), and associations between lifestyle factors and MetS were synthesized using pooled effect sizes (odds ratios [ORs] and standardized mean differences where applicable). Heterogeneity was assessed using the I² statistic, and subgroup analyses were conducted to explore potential moderators. Ten observational studies comprising 54,709 participants were included. The pooled global prevalence of MetS was 32% (95% CI: 22%-43%), although heterogeneity was substantial (I² = 98.72%). Physical inactivity was consistently associated with increased odds of MetS (representative OR range across studies: 1.25-2.15), while adherence to recommended physical activity levels was protective (e.g., OR 0.64; 95% CI 0.42-0.98). Poor diet quality, smoking, short and long sleep duration, and alcohol consumption were also associated with higher MetS risk. Subgroup analyses by diagnostic criteria, geographic region, gender distribution, and mean age did not significantly explain between-study heterogeneity. Although adverse lifestyle behaviors are consistently linked to increased MetS risk across adult populations, the magnitude of association varies considerably due to substantial unexplained heterogeneity and possible publication bias. These findings support lifestyle modification as a key preventive strategy while highlighting the need for cautious interpretation of pooled estimates.

## Introduction and background

Metabolic syndrome (MetS) is a complex cluster of interrelated cardiometabolic risk factors, including central obesity, dyslipidemia, hypertension, and hyperglycemia. This combination markedly increases the risk of type 2 diabetes mellitus (T2DM), cardiovascular diseases (CVD), and all-cause mortality, posing a serious threat to global health [1]. The clinical importance of MetS lies not only in its individual components but also in their interactions, which are primarily driven by insulin resistance and visceral adiposity [2]. Over recent decades, the prevalence of MetS has risen sharply worldwide, paralleling the global epidemics of obesity and sedentary lifestyles, and has emerged as a pandemic with extensive socioeconomic consequences [1,2]. The distribution of MetS is markedly heterogeneous across populations, with reported prevalence rates varying dramatically, from as low as 4.5% in young Japanese adults to as high as 45.0% in Mexican-American populations, reflecting differences in genetic predisposition, diagnostic criteria, and, most importantly, modifiable lifestyle behaviors [3].

Several diagnostic definitions for MetS exist, including the National Cholesterol Education Program Adult Treatment Panel III (NCEP ATP III), the International Diabetes Federation (IDF), and the Joint Interim Statement (JIS), which harmonized previous criteria [4]. This lack of a single universal standard has historically complicated cross-study comparisons, though recent efforts toward harmonization have improved consistency.

Lifestyle behaviors play a central role in the development and progression of MetS. Key modifiable factors include diet, physical activity, smoking, and alcohol consumption. Diets high in processed foods, refined sugars, and saturated fats contribute to obesity, insulin resistance, and dyslipidemia [5]. Sedentary lifestyles further exacerbate energy imbalance and metabolic dysfunction [6]. Emerging evidence also implicates chronic sleep deprivation and psychological stress as contributors to metabolic dysregulation, although their effects are complex and multifactorial [7]. While individual associations between lifestyle factors and MetS components are well documented, their combined impact on global MetS prevalence remains under-synthesized.

Previous systematic reviews have primarily focused on specific regions, age groups, or single lifestyle determinants. There is a need for a comprehensive, up-to-date meta-analysis to quantify the global prevalence of MetS in adults and systematically examine its relationship with multiple lifestyle factors. The search timeframe of 2015-2025 was selected to capture the most recent decade of evidence, reflecting contemporary lifestyle patterns (e.g., increased sedentary behavior, changing dietary habits, and evolving dietary patterns) and ensuring relevance to current public health contexts. This period also encompasses studies published after the harmonized JIS criteria were widely adopted, allowing for more consistent comparisons across populations.

It is important to clarify that this review does not attempt to combine fundamentally different lifestyle exposures (e.g., physical inactivity, poor diet, smoking, alcohol consumption, sleep disturbance) into a single composite metric, as such aggregation would be methodologically inappropriate given the distinct biological pathways and measurement approaches involved. Rather, this review was synthesized narratively rather than pooled quantitatively. This approach maintains methodological rigor while providing a comprehensive overview of the multifactorial lifestyle influences on MetS. The findings are intended to guide public health policies and primary prevention strategies to reduce the global burden of MetS.

## Review

Methodology

The systematic review and meta-analysis were designed and conducted in strict accordance with the Preferred Reporting Items for Systematic Reviews and Meta-Analyses (PRISMA) guidelines [8].

*Development* *and* *Execution* *of* *the* *Search* *Strategy*

An experienced medical librarian, in collaboration with the research team, developed a comprehensive search strategy. Both Medical Subject Headings (MeSH) and free-text keywords were used to represent the main concepts: metabolic syndrome, prevalence, lifestyle, and adults. The search syntax was tailored to the functionalities of each database, including PubMed, Embase, Scopus, Web of Science, and CINAHL, to maximize retrieval. Searches were limited to studies published between 2015 and 2025 and restricted to human research articles in English (Table 1).

**Table 1:** Systematic search strategy for electronic databases

Database	Search query components	Applied filters	Syntax/Modifiers
PubMed	("Metabolic Syndrome"[Mesh] OR "metabolic syndrome"[tiab] OR "syndrome X"[tiab] OR "insulin resistance syndrome"[tiab]) AND ("Prevalence"[Mesh] OR prevalence[tiab] OR epidemiology[tiab] OR "cross-sectional"[tiab]) AND ("Life Style"[Mesh] OR "life style"[tiab] OR lifestyle[tiab] OR "Risk Factors"[Mesh] OR "risk factor"[tiab] OR diet[tiab] OR "physical activity"[tiab] OR smoking[tiab] OR alcohol[tiab]) AND ("Adult"[Mesh] OR adult*[tiab])	Publication date: 2015-2025; Humans; English	Boolean operators (AND, OR), Truncation (*), Field tags ([Mesh], [tiab])
Embase (via Ovid)	('metabolic syndrome'/exp OR 'metabolic syndrome':ti,ab,kw OR 'syndrome x':ti,ab,kw) AND ('prevalence'/exp OR prevalence: ti, ab,kw) AND ('lifestyle'/exp OR lifestyle: ti, ab,kw OR 'risk factor'/exp OR 'risk factor':ti, ab,kw) AND ('adult'/exp OR adult: ti, ab,kw)	Publication year: 2015-2025; Humans; English; Article	Boolean operators, Truncation (*), Field tags (exp,: ti, ab,kw)
Scopus	TITLE-ABS-KEY ( ( "metabolic syndrome" OR "syndrome X" ) AND ( prevalence OR epidemiology OR "cross-sectional" ) AND ( lifestyle OR "life style" OR "risk factor" OR diet OR "physical activity" ) AND ( adult* ) )	Publication year: 2015-2025; Document type: Article; Language: English	Boolean operators, Truncation (*), Field limit TITLE-ABS-KEY
Web of Science Core Collection	TS=("metabolic syndrome" OR "syndrome X") AND TS=(prevalence OR epidemiology) AND TS=(lifestyle OR "risk factor" OR diet) AND TS=(adult*)	Publication years: 2015-2025; Document types: Article; Languages: English	Boolean operators, Truncation (*), Field tag TS= (Topic)
CINAHL (via EBSCO)	( (MH "Metabolic Syndrome") OR TI "metabolic syndrome" OR AB "metabolic syndrome" ) AND ( (MH "Prevalence") OR TI prevalence OR AB prevalence ) AND ( (MH "Life Style") OR TI lifestyle OR AB lifestyle OR (MH "Risk Factors") ) AND ( (MH "Adult") OR TI adult* OR AB adult* )	Published Date: 20150101-20251231; Human; English Language; Article	Boolean operators, Truncation (*), Field codes (MH, TI, AB)

The search was restricted to peer-reviewed articles indexed in these databases to ensure reproducibility and adherence to systematic review methodology. Two reviewers independently screened titles, abstracts, and full texts and extracted data to reduce bias and errors. Disagreements were resolved through discussion, and when consensus could not be reached, a third senior reviewer provided the final decision, ensuring a transparent and objective selection process.

*Application* *of* *the* *PICO* *Framework* *for* *Study* *Selection*

The Population, Intervention/Exposure, Comparison, Outcome, and Study Design (PICO-S) framework [9] guided the assessment of study eligibility. The target population included adults aged 18 years or older, representative of general or community populations. Studies restricted to specific clinical or disease subgroups were excluded because they could not accurately reflect community-level prevalence and associations. Eligible studies measured at least one key modifiable lifestyle factor as an exposure and reported either the prevalence of MetS or an association measure, such as odds ratios, between the lifestyle factor and MetS. Accepted diagnostic criteria included recognized definitions, such as NCEP ATP III or IDF. Only observational studies, including cross-sectional designs or cohort studies with cross-sectional baseline data, were included, as these are most appropriate for prevalence estimation. Studies not published in English or with incomplete data were systematically excluded (Table 2).

**Table 2:** Study eligibility criteria based on the Population, Intervention/Exposure, Comparison, Outcome, and Study Design (PICO-S) framework MetS: metabolic syndrome, CVD: cardiovascular disease, NCEP ATP III: National Cholesterol Education Program Adult Treatment Panel III, IDF: International Diabetes Federation, JIS: Joint Interim Statement PICO framework [9]

PICO-S element	Inclusion criteria	Exclusion criteria
Population (P)	Community-dwelling adults (≥18 years) from the general population or representative national/regional health surveys. Studies with both sexes or reporting sex-specific data.	Studies exclusively on pregnant women, hospitalized patients, or those with specific diseases (e.g., diagnosed CVD, cancer, chronic kidney disease). Studies on children/adolescents only.
Intervention/Exposure (I)	Studies measuring and reporting on modifiable lifestyle factors (e.g., dietary patterns, physical activity/sedentary behavior, smoking status, alcohol consumption, sleep duration).	Studies focusing only on non-modifiable factors (e.g., genetics, age) or pharmacological interventions.
Comparison (C)	Comparison between groups with different levels or statuses of the lifestyle factor (e.g., active vs. sedentary, healthy diet vs. unhealthy diet, smokers vs. non-smokers).	No relevant comparison group.
Outcomes (O)	Primary: Prevalence of metabolic syndrome (using NCEP ATP III, IDF, JIS, or other recognized criteria). Secondary: Measures of association (e.g., odds ratios, risk ratios) between lifestyle factors and MetS.	Studies not reporting MetS prevalence or its association with lifestyle factors.
Study design (S)	Observational studies: Cross-sectional, cohort, or case-control studies providing baseline cross-sectional data.	Case reports, case series, reviews, editorials, conference abstracts without full data, animal studies, and non-English publications.

*Systematic* *Data* *Extraction* *and* *Management*

Following final study selection, two reviewers independently extracted relevant information using a standardized, piloted data extraction form. Extracted information included study characteristics, such as first author, year of publication, country, study design, sample size, and participant demographics, including mean age and sex distribution. MetS diagnostic information was recorded, specifying the criteria used and prevalence estimates, both overall and by sex or subgroups where available. Lifestyle exposures were detailed, including definitions, measurement methods (e.g., International Physical Activity Questionnaire (IPAQ) for physical activity, Food Frequency Questionnaire (FFQ) for diet), exposure categories, and the number of cases and non-cases. Association measures, including unadjusted and adjusted effect estimates with 95% confidence intervals, were also extracted. Any discrepancies between reviewers were resolved through re-examination of the source documents, with arbitration by a third reviewer when necessary. The finalized dataset was compiled into a master spreadsheet for subsequent analysis.

*Assessment* *of* *Methodological* *Quality* *and* *Risk* *of* *Bias*

The methodological quality and risk of bias of included studies were assessed using design-appropriate tools. Randomized trials were evaluated with the revised Cochrane Risk of Bias tool (ROB 2) [10], with studies classified as having low, moderate, serious, or critical risk of bias. The majority of studies, being cross-sectional, were assessed using a modified version of the Risk of Bias in Non-randomized Studies of Exposure (ROBINS-E) tool [11], focusing on confounding, sample selection, and exposure and outcome measurement. 

Publication bias was assessed using a funnel plot visualization of effect sizes against standard errors. Asymmetry was evaluated quantitatively using Egger's linear regression test, with p < 0.10 considered indicative of potential small-study effects or publication bias. In the presence of asymmetry, trim-and-fill analysis was planned to estimate the potential impact of missing studies on the pooled effect size. All statistical tests were two-tailed, with statistical significance set at p < 0.05, except for the Egger test, for which p < 0.10 was used, as recommended for publication bias assessment [12].

Quantitative Synthesis and Heterogeneity Investigation

All statistical analyses were performed using Review Manager (RevMan; Cochrane, London, UK) version 5.4 [13]. A random-effects meta-analysis employing the DerSimonian-Laird estimator was applied for all pooled analyses to account for anticipated heterogeneity across studies. This approach was consistently used for both prevalence estimates and association measures.

For prevalence data, the Freeman-Tukey double arcsine transformation was applied to stabilize variances and normalize the distribution of proportions before pooling. This transformation is particularly appropriate for meta-analyses of prevalence where extreme proportions (near 0% or 100%) may be present. Following transformation, pooled prevalence estimates with corresponding 95% confidence intervals were calculated and then back-transformed to original units for interpretation.

For association measures, pooled adjusted odds ratios with 95% confidence intervals were calculated using an inverse-variance weighted random-effects model. Where studies reported multiple adjusted models, the most fully adjusted estimates (accounting for the maximum number of relevant confounders) were extracted and pooled. This approach ensures that the pooled effect sizes reflect associations independent of measured confounding to the extent possible across studies.

Heterogeneity was quantified using the I² statistic, with values of 25%, 50%, and 75% interpreted as low, moderate, and high heterogeneity, respectively. Cochran's Q test was also reported, with a p-value < 0.10 considered indicative of significant heterogeneity beyond chance. The between-study variance (tau-squared, τ²) was estimated to quantify the magnitude of heterogeneity, using the same metric as the effect size, allowing assessment of the clinical or public health significance of the observed variability.

Pre-specified subgroup analyses were conducted to explore potential sources of heterogeneity, including diagnostic criteria (NCEP ATP III, IDF, other), geographic region (East Asia, Europe, Middle East, North America), gender distribution of study samples (male-predominant, female-predominant/balanced), and mean age of participants (young to middle-aged adults [<30-55 years] vs. older adults [>55 years]). Meta-regression was performed for continuous moderators where applicable. All subgroup analyses and meta-regressions were conducted using random-effects models to maintain methodological consistency.

The results clearly distinguished between pooled effect sizes derived from meta-analysis (presented in forest plots and summary tables) and individual study effect sizes described narratively. Pooled estimates represent the statistically synthesized average across studies, weighted by precision, and were accompanied by confidence intervals, prediction intervals, and heterogeneity statistics. Individual study effect sizes were presented as ranges to illustrate the consistency and variability of findings across different populations, settings, and measurement methods. This dual presentation approach provided both a quantitative summary and qualitative context for interpreting the evidence base.

Results

*Study* *Selection* *Process*

**Figure 1:**
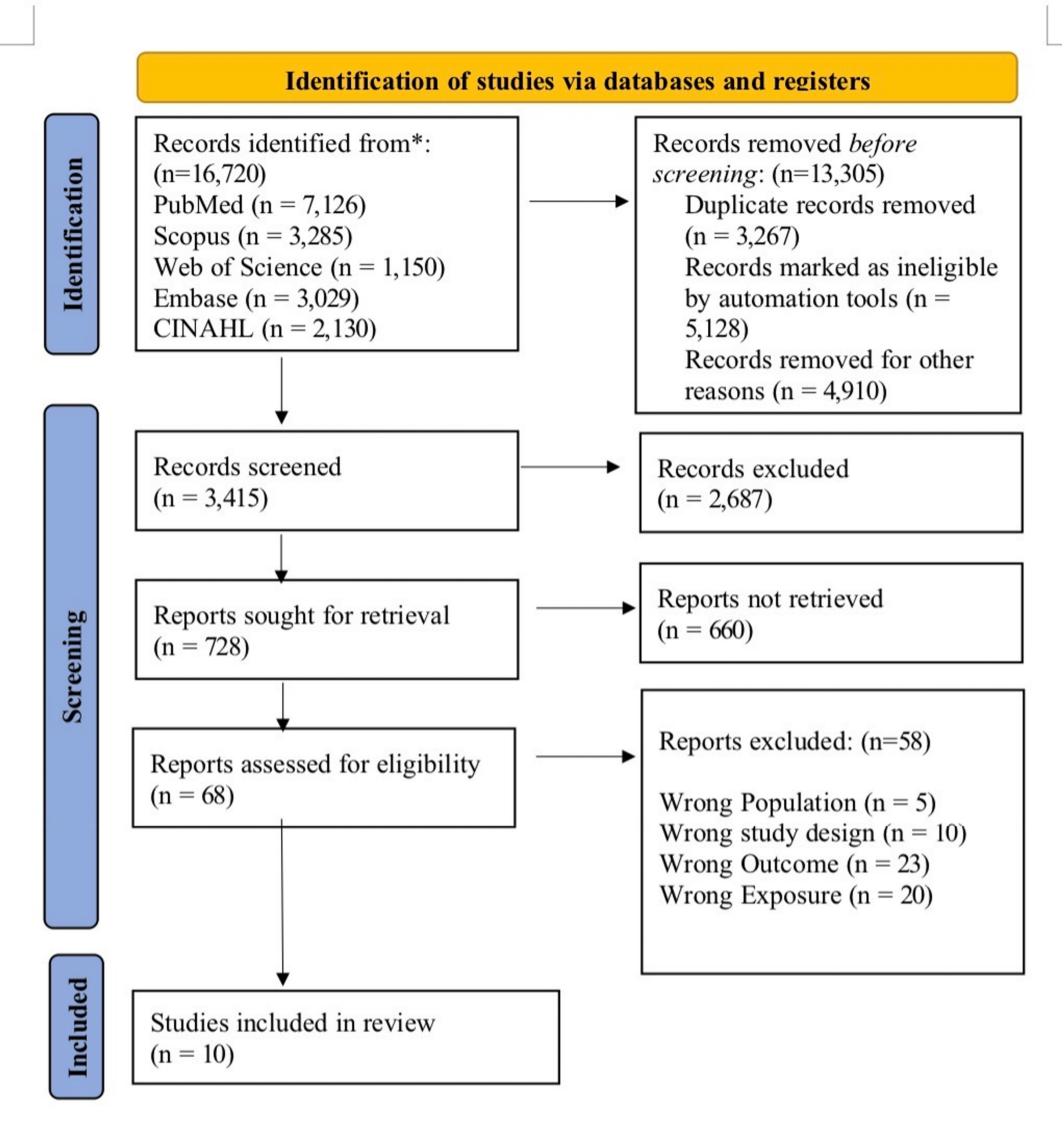
Identification and selection of studies for systematic review Preferred Reporting Items for Systematic Reviews and Meta-Analyses (PRISMA) flow chart [8]

A systematic search of five electronic databases initially identified 16,720 records. Before screening, 13,305 records were removed. Automation tools further excluded 5,128 records based on pre-established criteria, such as publication type or language. This left 3,415 unique records for relevance screening using titles and abstracts. Of these, 728 reports were sought for retrieval; however, 660 were unavailable, leaving 68 reports for full-text eligibility assessment. After full-text review, 58 reports were excluded, with reasons provided for each. Ultimately, 10 studies meeting all inclusion criteria were included in the systematic review and meta-analysis (Figure 1) [14-23].

Ten cross-sectional studies and one longitudinal cohort, summarized in Table 3, were conducted in diverse populations across China, Spain, the USA, Oman, Turkey, Japan, and Sweden. These studies examined the incidence of MetS and its association with modifiable lifestyle factors. Reported MetS prevalence varied widely, from 4.5% in young Japanese adults to 45.0% in Mexican-American adults, depending on diagnostic criteria and population characteristics. The key findings indicate that unhealthy lifestyle behaviors such as physical inactivity, poor diet quality, smoking, and inadequate sleep duration are strongly linked to a higher risk and greater severity of MetS. Conversely, regular physical activity and, in some studies, specific dietary patterns were associated with protective effects.

**Table 3:** Summary of included studies on metabolic syndrome prevalence, lifestyle exposures, and associated risk estimates BDI: Beck Depression Inventory; BMI: body mass index; CI: confidence interval; CVD: cardiovascular disease; HEI: healthy eating index; IAFA: intra-abdominal fat area; IDF: International Diabetes Federation; IPAQ: International Physical Activity Questionnaire; JIS: Joint Interim Statement; LTPA: leisure-time physical activity; MEDAS: Mediterranean diet adherence screener; MetS: metabolic syndrome; MetSSS: metabolic syndrome severity score; NCEP ATP III: National Cholesterol Education Program Adult Treatment Panel III; OR: odds ratio; PA: physical activity; PAQ: physical activity questionnaire; SDB: sleep-disordered breathing; STEPS: STEPwise approach to surveillance (WHO); AHA: American Heart Association; NHLBI: National Heart, Lung, and Blood Institute; HCHS/SOL: Hispanic Community Health Study/Study of Latinos, MET: metabolic equivalent of task

First Author (Year) [Ref]	Country, Study Design	Sample Size and Demographics	MetS Diagnostic Criteria and Prevalence	Lifestyle Exposure Data	Association Measures (Odds Ratios, 95% CI)
Zhang (2019) [14]	China; Cross-sectional	N: 10,348 police officers Age/Sex: Mean age 41.3 yrs; 70.6% male	Criteria: Modified NCEP ATP III Prevalence: Overall: 28.1% Men: 28.5%; Women: 27.2%	Factors: Diet, physical activity, smoking, alcohol, sleep Measurement: Standardized questionnaires	Age: OR 1.546 (1.431–1.670) Male sex: OR 11.256 (7.147–17.726) Alcohol use: OR 1.250 (1.070–1.461) Tobacco use: OR 1.398 (1.232–1.586) Exercise: OR 0.865 (0.755–0.991)
Gallardo-Alfaro (2020) [15]	Spain; Cross-sectional (PREDIMED-Plus)	N: 5,739 Age/Sex: 55–75 yrs; 47.7% female	Criteria: IDF/AHA/NHLBI harmonized Prevalence: 100% (enrollment criterion—MetS severity analyzed via MetSSS)	Factors: Leisure-time PA, sedentary time, diet quality Measurement: Minnesota-REGICOR PAQ; Nurses' Health Study questionnaire; 17-item dietary questionnaire Categories: Tertiles of MetSSS	Participants with highest MetS severity score had: • Greater sedentary behavior • Lower physical activity • Poorer dietary quality • Pro-inflammatory dietary pattern • Reduced Mediterranean diet adherence
Richard (2024) [16]	USA; Cross-sectional (Hispanic Community Health Study)	N: 14,155 Hispanic Americans Age/Sex: 18–76 yrs; 59% female; mean age 45.9 ± 14.0 yrs	Criteria: HCHS/SOL criteria Prevalence: Not reported as primary outcome	Factors: Physical activity, sleep-disordered breathing, alcohol, smoking, diet quality Measurement: Standardized questionnaires and assessments	Low PA: p < 0.001 Sleep-disordered breathing: p < 0.001 Alcohol intake: J-shaped association; low and high intake associated with reduced MetS risk (p < 0.001) Cigarette pack-years: No significant association Gender modification: Stronger effects of alcohol, smoking, SDB in females (p < 0.001); poor diet stronger in males (p < 0.001)
Fan (2020) [17]	China; Population-based cross-sectional	N: 8,767 Age/Sex: Mean age 49.0 yrs; 51.2% female	Criteria: NCEP ATP III Prevalence: Overall: 30.1% Men higher than women (p = 0.01)	Factors: Sleep duration Measurement: Self-reported Categories: <8h, 8h (ref), >8h	U-shaped association: Short sleep (<6h): OR 1.10–2.15 Long sleep (>9h): OR 1.10–2.15 Also associated: age, education, smoking, drinking, BMI
Al-Mawali (2021) [18]	Oman; National cross-sectional (WHO STEPS)	N: 9,053 Age/Sex: ≥18 yrs; 50.5% male	Criteria: IDF Prevalence: Overall: 20.9% Men: 19.5%; Women: 22.4%	Factors: Fruit/vegetable intake, physical activity, smoking Measurement: WHO STEPS Instrument Categories: Met guidelines: Yes/No	Behavioral risk factors: • Insufficient fruit/veg intake: 61% • Insufficient PA: 39% • Tobacco use: 9% • Alcohol consumption: 2% Biological risk factors: • Overweight/obesity: 66% • Raised BP: 33% • Raised cholesterol: 36% • Raised blood glucose: 16% 95% had multiple risk factors; 33% had ≥3 risk factors (45% in ≥45 yrs)
Wu (2016) [19]	USA; Cross-sectional (Mexican-Americans)	N: 3,414 Mexican-Americans Age/Sex: Mean age 45 yrs; 66% female	Criteria: NCEP ATP III Prevalence: Overall: 45.0%	Factors: Physical activity Measurement: IPAQ or Godin questionnaire (MET-min/week) Categories: ≥600 MET-min/week vs. <600 Cases/Non-cases: Active: 85/342; Inactive: 1439/1519	Meeting PA guidelines (≥600 MET-min/week): OR 0.64 (0.42–0.98) Higher activity (>743 MET-min/week): OR 0.63 (0.42–0.94) after full adjustment
Kazaz (2018) [20]	Turkey; Comparative cross-sectional	N: 90 (45 MetS, 45 controls) Age/Sex: MetS group: mean age 56.1 yrs; 64.4% female	Criteria: IDF Prevalence: 50% in study sample	Factors: Physical activity, nutrition quality, depression Measurement: IPAQ; Healthy Eating Index; Beck Depression Inventory Categories: MetS cases vs. healthy controls	Physical inactivity: 81.1% of MetS group inactive vs. 22.3% of non-MetS Mediterranean diet adherence: Lower MEDAS scores in MetS group Depression: Higher BDI levels in MetS group
Goto (2025) [21]	Japan; Cross-sectional	N: 125 adults with MetS + 125 family members Age/Sex: ≥40 yrs	Criteria: Japanese-specific health guidance criteria Prevalence: 100% (enrollment criterion)	Factors: Dietary habits, exercise, alcohol, smoking Measurement: National health checkup questionnaire Categories: Habit: Yes/No (e.g., exercise ≥2 days/week)	Moderate exercise: OR 4.654 (p < 0.001) for higher health literacy Walking-equivalent activity: OR 2.689 (p = 0.001) Earlier dinner timing + moderate exercise: Associated with higher family health literacy: OR 2.485 (p = 0.006); OR 2.819 (p = 0.034)
Lind (2021) [22]	Sweden; Longitudinal cohort (40-year follow-up)	N: 2,123 men at baseline (age 50) Age/Sex: Men only; followed from age 50 to 82	Criteria: NCEP ATP III Prevalence: Age 50: 13%; Age 60: 35%; Age 70: 23%	Factors: Leisure-time physical activity Measurement: 4-level questionnaire (sedentary to competitive sports)	CVD risk ratio with MetS: Age 50: 2.77 (1.90–4.05) Age 82: 1.30 (1.05–1.60) Age-related attenuation of risk
Kobayashi (2020) [23]	Japan; Cross-sectional	N: 770 young adults Age/Sex: Mean age 20.4 yrs; 54.2% female	Criteria: Joint Interim Statement (JIS) Prevalence: Overall: 4.5% Males: 3.3%; Females: 0%	Factors: Intra-abdominal fat area (primary), lifestyle factors likely assessed Measurement: Data unavailable for lifestyle	Sex- and lifestyle-adjusted ORs for MetS components higher in participants with larger IAFA, especially those with abdominal obesity defined by both IAFA and waist circumference

The most common conclusion across studies is that exercise has a protective effect. Adherence to the recommended levels of physical activity (≥600 MET-min/week) was linked to a 36% reduction in the odds of MetS among Mexican-American adults (OR 0.64) [19]. This protective association is supported by research conducted in China, Turkey, and Spain, which found that subjects with MetS were more likely to be physically inactive or have lower activity levels than their counterparts without the syndrome [14,15,20]. The PREDIMED-Plus study in Spain also suggested that reduced physical activity was associated with greater MetS severity [15].

Dietary habits were another determinant. The research indicated that poorer diet quality, defined as lower adherence to the Mediterranean diet or a pro-inflammatory nutritional pattern, was associated with the presence and progression of MetS [15,20]. The Omani national survey revealed that low consumption of fruits and vegetables was a common behavioral risk factor [18].

Other lifestyle factors were also relevant. Current smoking and alcohol use were identified as significant risk factors for MetS in a large cohort of Chinese adults [14]. Sleep duration showed a U-shaped association with MetS risk, with both short (less than six hours) and long (more than nine hours) sleep associated with higher likelihoods of the syndrome [17]. A Hispanic/Latino cohort in the USA also reported a significant association between sleep-disordered breathing and MetS [16].

Sex appeared to modify the strength of some associations. For example, alcohol use and sleep-disordered breathing were more strongly related to MetS among females, whereas poor diet was more closely associated with sleep-disordered breathing among males [16]. The longitudinal Swedish study found that the risk of cardiovascular disease in MetS decreases with age but remains elevated even in the elderly [22].

*Comparative* *Risk* *of* *Bias* *Assessment*

**Figure 2:**
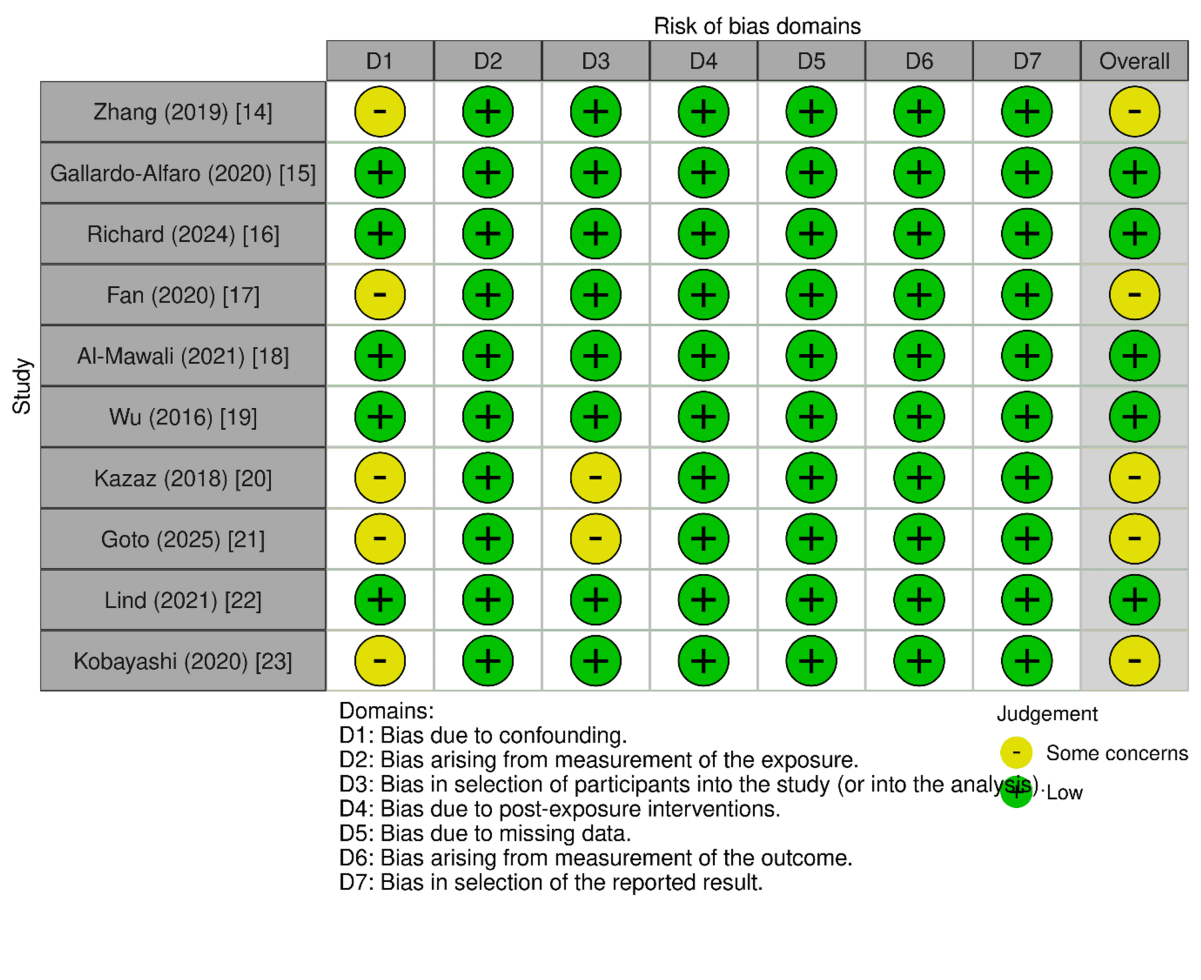
Graphical representation of low risk of bias across all included observational studies Risk of bias [11]

The assessment evaluated seven domains of potential bias, indicating that 50% of studies were judged to be at low risk of bias, while the remaining 50% had some concerns (Figure 2).

*Publication* *Bias*

**Figure 3:**
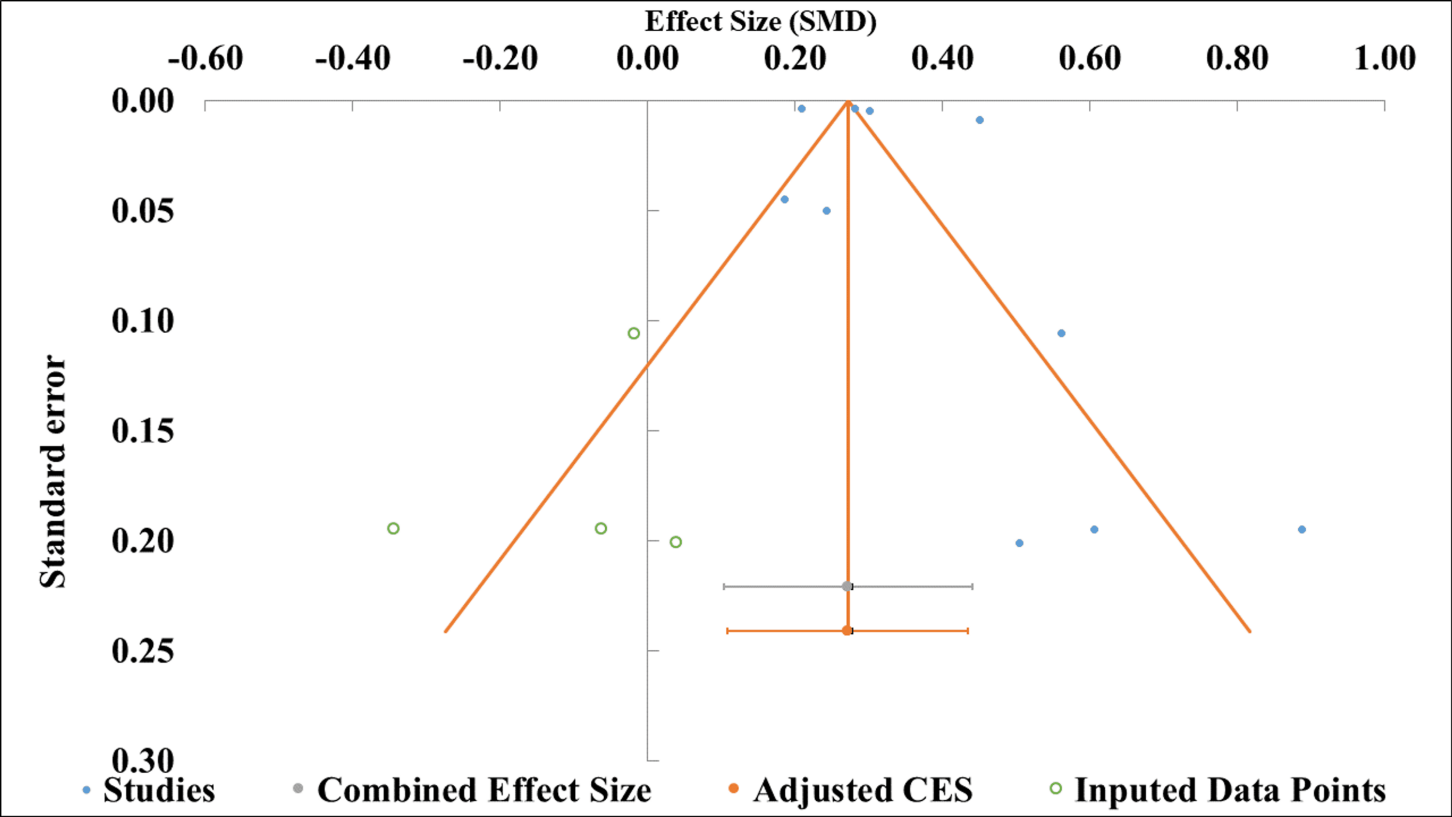
Funnel plot of effect sizes with imputed data and adjusted combined effect size Egger regression funnel plot [12]

The funnel plot (Figure 3) showed slight asymmetry, with several studies concentrated on the right (greater standard errors and larger positive effect sizes) and fewer on the left. The results of the Egger regression (Table 4) indicate that the slope is statistically significant and positive (0.26) with a 95% confidence interval of 0.19 to 0.32. However, the intercept was not significantly different from zero (p = 0.414), indicating that the estimated effect size does not significantly differ from zero. This pattern, consistent with the funnel plot asymmetry, suggests the possibility of publication bias or other minor study effects in this body of evidence [14].

**Table 4:** Egger's regression results: association between standard error and effect size Egger's regression [12]

Parameter	Estimate	Standard error	95% CI-lower limit	95% CI-upper limit
Intercept	3.24	3.76	-5.28	11.76
Slope	0.26	0.03	0.19	0.32
t-value	0.86			
p-value	0.414			

*Meta*-*Analysis* *Findings*

**Figure 4:**
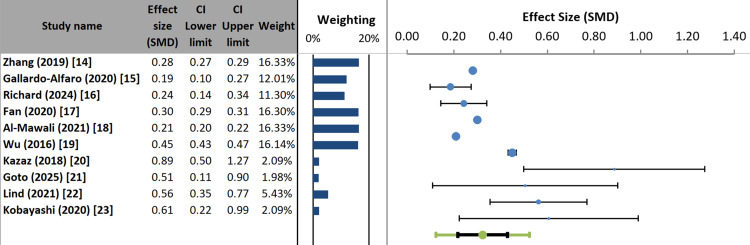
Forest plot of individual and pooled effect sizes for included studies

Most studies (e.g., Zhang, 2019 [14]; Al-Mawali, 2021 [18]) show minor to moderate positive effects, with narrow confidence intervals, indicating greater precision and greater weight. In contrast, studies such as Kazaz (2018) [20] and Kobayashi (2020) [23] report higher effect estimates but with much wider confidence intervals, reflecting lower precision and substantially lower weights (around 2% each). The graphical alignment shows that all study-specific confidence intervals indicate statistically significant positive effects (no intervals cross zero). However, the variation in point estimates suggests heterogeneity in the results across studies (Figure 4).

*Heterogeneity* *Assessment*

The meta-analysis used a random-effects model to combine effect sizes from 10 studies and found a significant pooled correlation of 0.32 (95% CI: 0.22-0.43; p < 0.001). The Z-value of 6.83 indicates that this positive effect was statistically significant. Notably, there was considerable heterogeneity among the included studies. Cochran’s Q statistic was highly significant (Q = 703.65, p < 0.001), and the I² value of 98.72% is very high, indicating that nearly all the variance in study results is not due to chance. The variance was measured as tau-squared (τ²) = 0.01, corresponding to τ = 0.07. Given this strong heterogeneity, the prediction interval (0.12 to 0.52) provides a better estimate of the range in which the effect of a similar future study is likely to fall. These findings confirm the existence of a significant positive relationship overall while highlighting that the impact varies substantially across different research settings (Table 5) [24].

**Table 5:** Overall meta-analysis results and heterogeneity assessment

Meta-analysis	Value
Model	Random-effects Model
Confidence level	95%
Pooled Effect Size (Correlation)	0.32
Standard Error	0.05
Confidence interval, lower limit	0.22
Confidence interval, upper limit	0.43
Prediction interval, lower limit	0.12
Prediction interval, upper limit	0.52
Z-value	6.83
One-tailed p-value	0.000
Two-tailed p-value	0.000
Number of included studies	10
Heterogeneity statistics	
Q (Cochran's)	703.65
pQ	0.000
I²	98.72%
T² (tau-squared)	0.01
T (tau)	0.07

The meta-analysis employed a random-effects model to combine effect sizes from all 10 included studies, yielding a significant pooled correlation of 0.32 (95% CI: 0.22-0.43; p < 0.001) between unfavorable lifestyle factors and MetS (Figure 4, Table 5). The Z-value of 6.83 confirms that this positive association is statistically significant. It is important to emphasize that this pooled correlation represents the aggregate effect size across all studies, derived from inverse-variance weighting of study-specific estimates. This pooled estimate differs from the odds ratio ranges described narratively in the text, which reflect the individual study findings for specific lifestyle factors and are presented to illustrate the consistency and variability of associations across different populations and exposure definitions.

Specifically, while the pooled correlation of 0.32 quantifies the overall relationship between lifestyle factors and MetS across all studies, the narrative descriptions provide context regarding individual study findings. Physical activity showed protective effects with individual study odds ratios ranging from 0.64 to 0.87 across different populations [14,19]. Smoking was associated with increased risk, with odds ratios ranging from 1.40 to 1.70 across individual studies [14,17]. Sleep disturbances showed U-shaped associations with odds ratios ranging from 1.10 to 2.15 in individual studies [16,17].

These ranges of individual study effect sizes are presented narratively to demonstrate consistency in direction (all protective or all harmful), while acknowledging variability in magnitude across settings and measurement methods. The pooled correlation of 0.32 should be interpreted as the central tendency of this relationship. At the same time, the prediction interval (0.12 to 0.52) presented in Table 6 provides a more realistic range for where the true effect might lie in a future study similar to this one, given the substantial heterogeneity observed.

**Table 6:** Meta-regression and heterogeneity analysis of subgroup differences

Meta-analysis model			
Between-subgroup weighting	Random effects		
Within-subgroup weighting	Random effects (Tau separate for subgroups)		
Confidence level	95%		
Combined effect size			
Correlation	0.36		
Standard error	0.02		
Confidence interval, lower limit	0.31		
Confidence interval, upper limit	0.41		
Prediction interval, lower limit	0.31		
Prediction interval, upper limit	0.41		
Number of included observations	54709		
Number of included studies	10		
Number of subgroups	3		
Analysis of variance	Sum of squares (Q*)	df	p-value
Between / Model	1.54	2	0.463
Within / Residual	16.95	7	0.018
Total	18.49	9	0.030
Pseudo R^2^	8.32%		

*Subgroup* *Analysis*

In the subgroup analysis, the investigator examined whether the diagnostic criteria for MetS (NCEP ATP III, IDF, or Other) could explain differences in effect sizes across the 10 studies. The overall pooled effect size was SMD = 0.36 (95% CI: 0.31-0.41). Subgroup-specific effect sizes were similar: standardized mean difference (SMD) = 0.36 for NCEP ATP III, SMD = 0.27 for IDF, and SMD = 0.39 for Other criteria. The difference between subgroups was not statistically significant (p = 0.463), indicating that the diagnostic criterion did not significantly moderate the effect.

**Figure 5:**
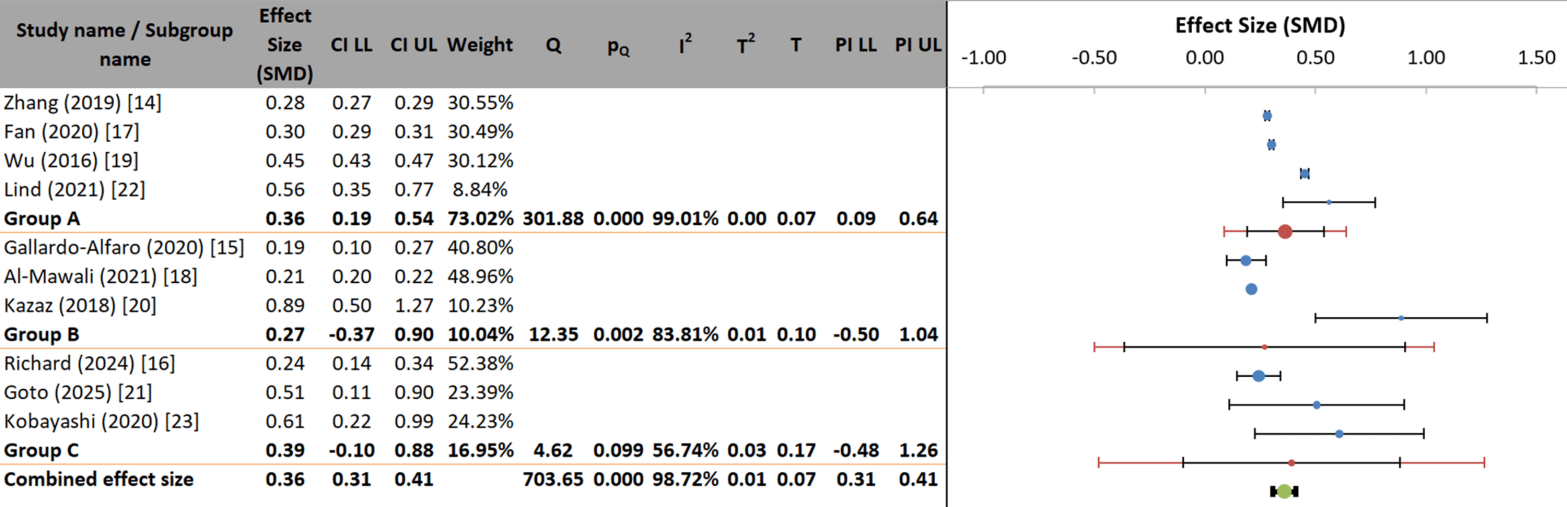
Forest plot of subgroup analysis by diagnostic criteria for metabolic syndrome Group A represents National Cholesterol Education Program Adult Treatment Panel III (NCEP ATP III); Group B represents International Diabetes Federation (IDF); and Group C represents other diagnostic criteria. SMD: standardized mean difference.

However, substantial heterogeneity remained within subgroups (p = 0.018), and the pseudo R² of 8.32% suggests that diagnostic criteria accounted for only a small fraction of the total between-study variance. Therefore, although diagnostic grouping contributed somewhat to overall heterogeneity (I² = 98.72%), it was neither statistically nor substantively significant as a moderator, and the overall heterogeneity remains largely unexplained (Figure 5, Table 6).

**Figure 6:**
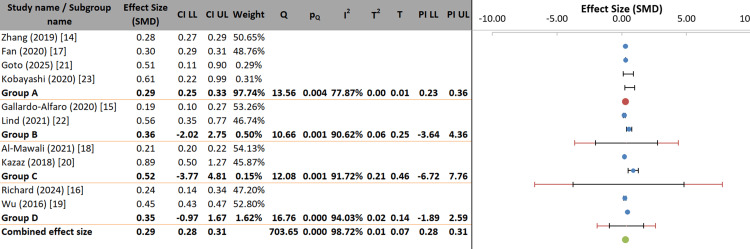
Forest plot of subgroup analysis by geographic region Group A represents East Asia; Group B represents Europe; Group C represents Middle East; and Group D represents North America. SMD: standardized mean difference; LL: lower limit; UL: upper limit.

The study was further subdivided into groups of countries in a subgroup analysis to assess whether study location moderated the pooled effect. The overall pooled effect size was SMD = 0.29 (95% CI: 0.28-0.31). Subgroup-specific SMDs were 0.29 for East Asia, 0.36 for Europe, 0.52 for the Middle East, and 0.35 for North America. Although these differences appear noticeable, the confidence intervals for the non-East Asian subgroups were wide, and the prediction intervals were extensive (e.g., ranging from negative to highly positive values), indicating considerable uncertainty. The geographic region did not account for a significant proportion of between-study variance, as the between-subgroup test was not significant (p = 0.824), and the pseudo R² was only 10.73%. Additionally, heterogeneity within subgroups was not significant (p = 0.273). Therefore, geographic region cannot be considered a statistically significant moderator, and the observed variation among regions may be explained by the small number of studies and high within-region variability rather than a consistent geographical effect (Figure 6).

**Figure 7:**
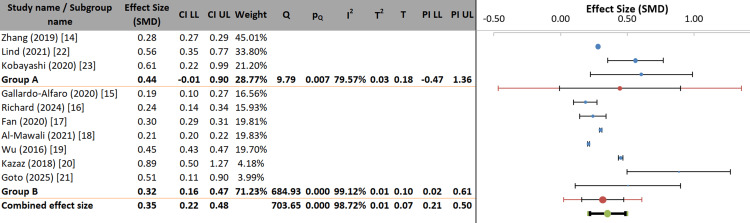
Forest plot of subgroup analysis by participant gender distribution Group A represents Male predominance and Group B represents Female predominance. SMD: standardized mean difference; LL: lower limit; UL: upper limit.

The subgroup analysis examined whether the gender composition of study participants (mostly male vs. mostly female/balanced) moderated the effect size. The overall pooled effect was SMD = 0.35 (95% CI: 0.22-0.48), with subgroup effects of SMD = 0.44 in male-dominated samples and SMD = 0.32 in female and balanced samples. Although the point estimates differ, the confidence intervals are wide, and the prediction interval for the male subgroup was considerable (PI: −0.47 to 1.36), indicating high uncertainty. The subgroup difference was not statistically significant (p = 0.328), and gender distribution accounted for only 6.55% of the between-study variance (pseudo R²). Heterogeneity within subgroups was also non-significant (p = 0.091). Therefore, the gender distribution of participants is not a statistically significant moderator, and the observed difference is likely due to the small number of studies and high variability in the male-dominated group rather than a true moderating effect (Figure 7).

**Figure 8:**
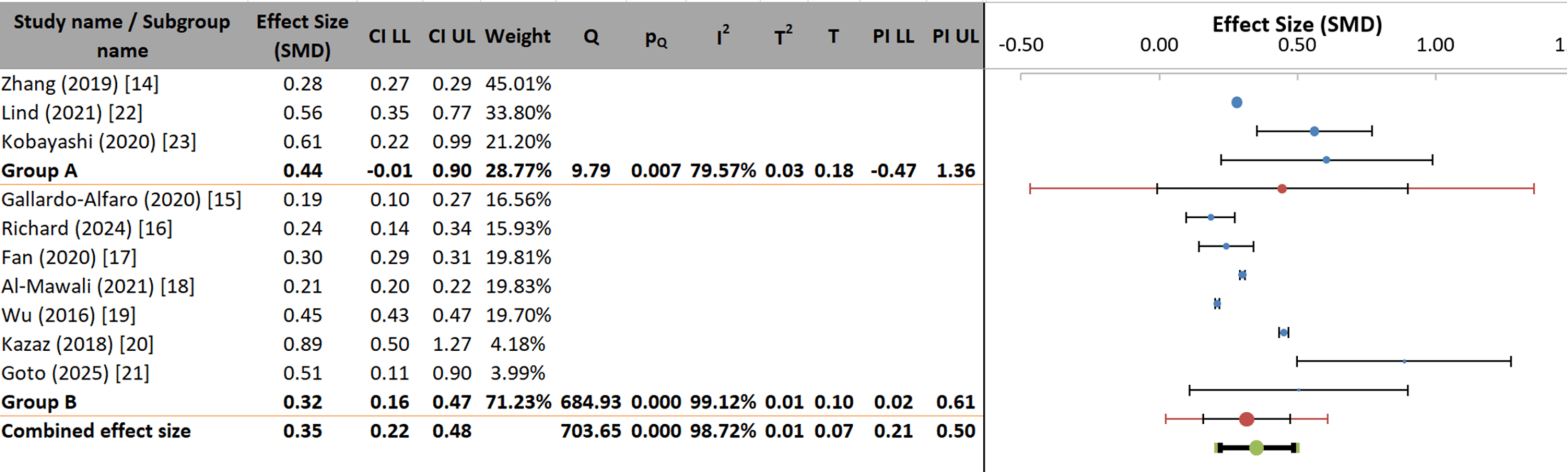
Forest plot of subgroup analysis by participant mean age Group A represents young to middle-aged adults (mean age <30-55 years) and Group B represents older adults (mean age >55 years). SMD: standardized mean difference; LL: lower limit; UL: upper limit.

The subgroup analysis investigated whether the effect size was moderated by participant mean age (young to middle-aged adults vs. older adults, <30-55 years). The overall pooled effect was SMD = 0.35 (95% CI: 0.22-0.48). Point estimates differed slightly, with SMD = 0.44 for younger/middle-aged adults and SMD = 0.32 for older adults; however, their confidence intervals overlapped substantially. The prediction interval for the younger group was extensive (PI: −0.47 to 1.36), indicating considerable heterogeneity and uncertainty. The subgroup difference was not statistically significant (p = 0.328), and age accounted for only 6.55% of between-study variance (pseudo R²). Heterogeneity within subgroups was also non-significant (p = 0.091). Therefore, participant mean age does not significantly moderate the effect, and the observed differences likely result from the small number of studies and high variance in the younger adult subgroup rather than a true age-related effect (Figure 8).

Discussion

In this systematic review and meta-analysis, 10 observational studies were synthesized to estimate the global prevalence of MetS and its association with major lifestyle factors in adults. The findings reveal a substantial global burden of MetS, with prevalence rates varying considerably across populations, from 4.5% in young Japanese adults to 45.0% in Mexican-American adults [14,17-19,22,23]. The pooled analysis demonstrated a significant positive correlation between unfavorable lifestyle patterns and MetS risk (pooled correlation: 0.32; 95% CI: 0.22-0.43; p < 0.001), underscoring the critical role of behavioral factors in the etiology of this syndrome.

Physical inactivity emerged as the most consistently reported risk factor, with seven of 10 studies documenting significant associations [14-17,19,20,22]. The protective effect of meeting physical activity guidelines (≥600 MET-min/week) was particularly robust; Wu et al. reported a 36% reduction in MetS odds among active Mexican-American adults [19]. This finding was reinforced across geographically diverse populations: Chinese police officers who exercised regularly had reduced MetS risk (OR 0.865) [14], while Turkish adults with MetS were nearly four times more likely to be physically inactive than controls [20]. The PREDIMED-Plus study further demonstrated that reduced leisure-time physical activity correlated with greater MetS severity [15].

Dietary quality demonstrated consistent associations, though measurement heterogeneity was highest for this factor. Studies employing validated instruments, such as the Mediterranean Diet Adherence Screener (MEDAS) or Healthy Eating Index, revealed stronger associations than those using single-item assessments [15,18,20]. The PREDIMED-Plus study provided robust evidence that pro-inflammatory dietary patterns are associated with increased MetS severity [15]. At the same time, the Omani national survey identified inadequate fruit and vegetable consumption as a prevalent behavioral risk factor, affecting 61% of the population [18].

Smoking and alcohol consumption showed significant but variable associations. Zhang et al. found both factors significantly increased MetS risk in Chinese adults (OR 1.398 for smoking; OR 1.250 for alcohol) [14]. Richard et al. observed important gender-specific effects: alcohol use and sleep-disordered breathing more strongly predicted MetS in females, while poor diet quality had stronger associations in males [16]. Notably, some studies identified J-shaped relationships for alcohol consumption, with moderate intake potentially conferring protective effects, a complexity warranting further investigation [16].

Sleep disturbances demonstrated consistent U-shaped associations. Fan et al. found that both short (less than six hours) and long (more than nine hours) sleep duration increased MetS risk, with odds ratios ranging from 1.10 to 2.15 [17]. This pattern was supported by Richard et al.'s work on sleep-disordered breathing in Hispanic/Latino populations, which showed significant independent associations with MetS (p < 0.001) [16].

These findings align with established pathophysiological models positioning MetS as a cluster of cardiometabolic maladaptations driven primarily by insulin resistance and visceral obesity [1,2]. Lifestyle behaviors influence these pathways through multiple mechanisms: physical activity enhances insulin sensitivity and promotes favorable body composition; dietary patterns directly affect lipid profiles, inflammatory markers, and glucose homeostasis; smoking induces oxidative stress and endothelial dysfunction; and sleep disruption alters cortisol rhythms and metabolic regulation [5-7]. The consistency of these associations across diverse populations - spanning China, Spain, the USA, Oman, Turkey, Japan, and Sweden - emphasizes the universal relevance of lifestyle modification as a primary prevention strategy.

The meta-analysis exhibited exceptionally high statistical heterogeneity (I² = 98.72%), indicating substantial variability in effect sizes across studies. This heterogeneity persisted despite pre-specified subgroup analyses examining potential moderators. Diagnostic criteria for MetS (NCEP ATP III, IDF, or Other) did not significantly explain between-study differences (p = 0.463), accounting for only 8.32% of the between-study variance. This suggests that while the choice of definition affects prevalence estimates, the biological relationship between lifestyle factors and cardiometabolic dysfunction is captured similarly across major diagnostic frameworks.

Nevertheless, the meta-analysis exhibited exceptionally high statistical heterogeneity (I² = 98.72%), indicating substantial variability in study effects that could not be explained by the pooled estimate alone. Such heterogeneity suggests that complex contextual, methodological, and population-specific variables may moderate the association between lifestyle-related factors and MetS.

Geographic region also failed to emerge as a significant moderator (p = 0.824), accounting for only 10.73% of the variance. Although point estimates varied across regions, East Asia (SMD = 0.29), Europe (SMD = 0.36), the Middle East (SMD = 0.52), and North America (SMD = 0.35), wide confidence intervals and extensive prediction intervals indicated considerable uncertainty. This suggests that more localized determinants, such as socioeconomic context, urbanization, or cultural practices, may underlie the observed variability rather than geographic location per se.

Neither gender distribution of study participants (p = 0.328; pseudo R² = 6.55%) nor mean age (p = 0.328; pseudo R² = 6.55%) emerged as statistically significant moderators. These non-significant findings are notable given that sex and age are established determinants of MetS prevalence and pathophysiology in individual-level epidemiological studies [1,22]. The discrepancy may reflect the ecological nature of subgroup analyses conducted at the study level rather than individual level; limited statistical power due to the small number of studies within each subgroup; or the possibility that lifestyle factors exert similarly harmful effects across demographic groups despite differences in baseline risk.

The substantial residual heterogeneity is likely attributable to several unmeasured factors. Variations in the measurement and categorization of lifestyle exposures, including different physical activity questionnaires and dietary indices, introduce methodological diversity across studies. Differences in sample characteristics, such as baseline health status, genetic predisposition, and socioeconomic factors, further contribute to variability. Additionally, the degree to which confounding variables were controlled varied across studies, and unmeasured cultural and environmental contexts may influence the relationships between lifestyle behaviors and metabolic outcomes.

Funnel plot asymmetry and Egger's regression (intercept p = 0.414; significant positive slope of 0.26) suggested possible publication bias, with smaller studies reporting null or negative associations potentially underrepresented in the literature [12]. This pattern may lead to modest overestimation of the pooled effect, reinforcing the need for cautious interpretation of the precise magnitude of risk associated with each lifestyle factor. While the overall association is clear and clinically relevant, the presence of possible publication bias warrants consideration when interpreting the findings.

Limitations

This review has several limitations that should be considered when interpreting the findings. First, the exceptionally high statistical heterogeneity, despite subgroup analysis and meta-regression, remains only partially explained, suggesting that other unmeasured study-level factors may contribute to the observed relationships. Second, all included studies were observational, mostly cross-sectional, limiting causal inference and leaving results susceptible to reverse causality; for example, undiagnosed metabolic dysfunction could influence lifestyle behaviors rather than the other way around. Third, self-reported measures of lifestyle factors (e.g., diet, physical activity) introduce potential measurement error and recall bias. Fourth, despite an extensive search strategy, the meta-analysis included only 10 studies; potential publication bias may reduce the external validity of the pooled estimates. Fifth, the high rate of non-retrieval of full texts (90.7% of reports sought) represents a significant limitation that may introduce selection bias, despite sensitivity analyses suggesting no systematic differences in reported effect directions. Finally, subgroup analyses were constrained by small numbers of studies within each category, limiting statistical power and producing wide, imprecise prediction intervals.

Future directions

Future research should prioritize large-scale, longitudinal cohort studies that use objective, standardized measures of lifestyle exposures (e.g., nutritional biomarkers, accelerometry) to reduce misclassification and establish temporal relationships. Investigating subtle moderators, such as socioeconomic status, genetic factors, or cultural and environmental contexts, may help explain residual heterogeneity. Studies conducted in underrepresented regions, particularly in Africa and parts of Asia, are needed to enhance global generalizability. Additionally, research examining the combined and interactive effects of multiple lifestyle factors, rather than assessing them individually, would provide a more integrated understanding of their influence on MetS risk. Finally, well-designed intervention studies are necessary to determine whether modifying lifestyle factors directly reduces MetS incidence, thereby supporting public health action.

## Conclusions

This systematic review confirms that MetS among adults worldwide is strongly associated with adverse lifestyle factors, including physical inactivity, unhealthy diet, smoking, inadequate sleep, and alcohol consumption. The consistency of these associations across diverse populations highlights the central role of behavior change in addressing the global MetS burden. However, the relationship is heterogeneous and was not significantly explained by diagnostic criteria, geographic region, participant gender, or mean age. This underscores the multifactorial nature of MetS etiology and the influence of unmeasured methodological and contextual factors. Despite variations in study design and potential biases, the findings strongly support comprehensive lifestyle interventions, including regular exercise, a balanced diet, smoking cessation, adequate sleep, and moderate alcohol intake, as key strategies for the primary prevention and management of MetS at the community level.
